# Shame, Guilt, and Medical Error in Ann Patchett's *State of Wonder*

**DOI:** 10.1353/lm.2022.0030

**Published:** 2022

**Authors:** Luna Dolezal, Arthur Rose

**Keywords:** medical error, shame, guilt, Ann Patchett, second victim

## Abstract

Through exploring the relation between shame, guilt, and medical error in Ann Patchett's novel *State of Wonder* alongside author-physician Danielle Ofri's autobiographical reflections in her essay "Ashamed to Admit It: Owning up to Medical Error," this essay considers how fiction and medical nonfiction might contribute to an understanding of the experience of medical error and being a "second victim" from the point of view of the medical learner. We argue that *State of Wonder* expands the scope of Ofri's work on shame about medical error; the novel presents a more durational examination of an error's consequences in non-medical contexts.

Medical error can be a devastating experience for medical practitioners who are often called the "second victims" of medical mistakes.^1^ The emotional toll medical error takes on doctors is not well understood, with few studies investigating shame and/or guilt in response to making mistakes. This essay considers how fiction and medical nonfiction might contribute to this understanding, by exploring the relation between shame, guilt, and medical error in Ann Patchett's novel *State of Wonder* (2011) alongside Danielle Ofri's autobiographical reflections in her essay, "Ashamed to Admit It: Owning up to Medical Error," later reprinted as part of a chapter entitled "Burning with Shame" in *What Doctors Feel* (2013).

As Judith Fletcher and others have noted, *State of Wonder* recasts Joseph Conrad's *Heart of Darkness* within the neocolonial context of Big Pharma extractivism in the Amazon.^2^ *State of Wonder* describes the journey of Marina Singh, a former trainee doctor and now medical researcher, from Minnesota to Manaus, and then up the Rio Negro into the Brazilian Amazon. She is on a dual mission for her employer, the pharmaceutical company Vogel: uncover the details about the death of her colleague and friend, Anders Eckman, while also locating Dr. Annick Swenson, the task that initially sent Anders to the Amazon. Swenson is an elusive and enigmatic medic working for Vogel to develop a fertility drug from the bark of a rare tree guarded by a tribe called the Lakashi. In a striking parallel to Marlow, her literary predecessor, Marina's reason for this quest into the heart of darkness is to find a protegee of Enlightenment thinking recast as agent of extractive capital. In this case, the part of Kurtz is played by Swenson, whose pharmacological equivalent of Kurtz's ivory mission is the synthesis of[Other P-326] a lucrative drug that will delay the onset of menopause and prolong fertility into old age. While developing this fertility drug, Swenson inadvertently discovers an effective malaria preventative, which she is developing in secret. This larger political situation forms the backdrop to Marina's professional relationship with Swenson and her obsessive recollections of a medical error she committed while she was Swenson's student, a mistake that led her to give up medicine.

While Patchett's *State of Wonder* is ostensibly the story of a journey to a pharmaceutical research station in a remote corner of the Amazon, the novel provides a compelling account of medical error and its emotional profile. Marina blinded a baby in one eye during an emergency Caesarean while training to be an obstetrician. The shame, and associated guilt, arising from the incident take an enormous existential toll, shaping her life trajectory and her overall sense of self. After the incident, Marina quits medicine, and severs all ties with those who knew about the incident and the investigation that followed, to pursue a career in research with Vogel. While Marina's shame is never made explicit in the novel (the action of which takes place over a decade after the accident), the narrative nonetheless yields a powerful portrait of maladaptive shame and its consequences, providing important insights into the long-term affective toll that medical error can take on medical learners.

In this essay, we consider how *State of Wonder*'s fictional depiction of medical error nuances discussions of the shame experienced by medical learners, exemplified by Ofri's autobiographical account. Our contention is that *State of Wonder* offers an enriching text for researchers or practitioners who are concerned with investigating shame as a "sentinel emotional event."^3^ We begin by considering the account of medical error in *State of Wonder* and within the wider health research literature. Next we consider the complex relationship between shame and guilt using Helen Block Lewis's psychoanalytic theory. We then turn to Ofri's engagement with medical error in "Burning with Shame" to establish how her own difficult confession of a medical error event relates to her umbrage over *State of Wonder* as described in her 2011 *Lancet* review of the novel. We end by considering the question of "writing shame" and the parallel concern arising from the "shame of writing" to address the conspicuous absence of shame in Patchett's novel (Marina only reflects on her "guilt"), Ofri's self-reported "agony" in confessing her own shame experience, and the shame of writing manifested in the text that Patchett's novel resembles: Conrad's *Heart of Darkness*.[Other P-327]

## I. Medical Error

Making mistakes is a normal human experience. In the medical profession, however, making mistakes has historically been treated as an aberration. In her 2020 consideration of medical error, *When We Do Harm*, Ofri notes that, until fairly recently, "the general approach [was] to figure out what—or more often who—had malfunctioned and then fix that thing."^4^ In fact, medical culture places an emphasis on perfectionism, implicitly expecting doctors and other healthcare professionals to be free from normal human error.^5^ As a result, the medical profession often attracts high-achieving perfectionists who easily internalize this expectation of infallibility, developing little tolerance for mistakes or errors in practice.^6^ As Ofri muses in her earlier work, the "culture of perfection in medicine" leads to a binary conception of healthcare practitioners: "either you are an excellent doctor or you are a failure."^7^ However, medicine is an imperfect science and necessarily involves ambiguity in diagnosis and the inaccuracies and complexities of embodied human interaction. As Diane Aubin and Sharla King note, the standard of perfection is impossible to meet in health care due to a variety of reasons: daily opportunities to misdiagnose, misinterpret and miscalculate; rapid changes in medical knowledge; complex situations that are unpredictable and subject to rapid change; and the regular need for unplanned and uncertain decisions where there are no conclusive answers or solutions.^8^ Mistakes in medicine, then, are inevitable—especially when doctors are still in training.^9^

On her journey to find Swenson, Marina's thoughts obsessively return to her own sentinel emotional event as a trainee doctor under Swenson's tutelage. While an obstetrics and gynecology resident at the County Receiving Hospital in Baltimore, Marina attended a young mother who was experiencing a difficult labor. Because the patient wasn't dilating and the fetal heart rate was unstable, an emergency C-section became necessary. Although Swenson tells Marina to wait until she arrives to perform the procedure, Marina decides to defy Swenson's orders and perform the operation on her own after hours had passed and Swenson had not arrived.

The skin of the patient's belly was stretched to the point of startling thinness. … Marina remembered there was a sheen to it. She cut the skin, dug through the fat for the fascia. She had thought there was no time left. Her hands were working at triple speed, and there was the uterus. She thought that she was saving the[Other P-328] baby's life because she was so fast, but the instant she realized he was occiput posterior, looking straight up, the blade had caught his head right of center at the hairline, cutting until she stopped in the middle of his cheek … the scalpel slicing through the eye.^10^

Marina blinds the baby in one eye, leaving a scar across his face: "The specialists were already working but some things cannot be set to right" (70). She apologizes to the young mother and the baby's father, who accept her apology; the incident is resolved favorably after an investigation, and Marina is cleared by the hospital: "When all of it was over and the lawsuit was settled, she was allowed to go back. The patient had liked her, that was the hell of it. They had spent the whole night together. She wanted the settlement money but she didn't want Marina's head on a pike. She said that other than that one mistake she'd done a good job. That one mistake. So Marina was left to mete out a punishment for herself" (70). Absolution from the baby's parents and the medical establishment fail to alleviate Marina's painful emotions and negative self-conceptions associated with the error. She believes she is no longer good enough to be a trainee doctor: "She could not touch a patient, or face her classmates" (70).

When a medical error occurs, there is an obvious negative effect for the patient; they may be harmed, sometimes even fatally. Marina's case demonstrates, however, that healthcare professionals are also adversely affected by medical errors and it might be useful to recognize them as "second victims."^11^ A second victim can be understood as a healthcare professional "who is deeply affected and traumatized as a result of an adverse medical event."^12^ The absence of supportive supervisors and work environment can exacerbate this traumatization.^13^ Medical errors can lead to withdrawal, quitting (a specialty or the medical profession altogether), depression, and in severe cases even suicide. While strong emotional reactions and psychological distress in response to medical errors are widely acknowledged,^14^ there is, as William Bynum and Jeffrey Goodie point out, almost no research exploring the common emotional responses of shame and guilt in relation to them.^15^ Despite the culture of perfectionism in medicine creating, as Aubin and King observe, "a perfect ecosystem for growing shame when an error is introduced,"^16^ this lack of research is perhaps unsurprising since this culture makes admitting mistakes so difficult. Shame about mistakes is replicated in the relative silence on this topic within the research.[Other P-329]

## II. Shame and Guilt

Marina understands her response to the incident as guilt, rather than shame: "The great, lumbering guilt that slept inside of her at every moment of her life" (64). However, insofar as this "guilt" imbricates itself into "every moment" of Marina's life, it betrays certain qualities usually associated with shame. To appreciate this distinction, we need to address the roles that guilt and shame play in the affective landscape of medical error.^17^

Shame and guilt are both negative self-conscious emotions that arise as a result of a negative event, where one has made a mistake, transgressed a social norm, or is in the wrong in some way. Shame and guilt both involve a concern with how one is perceived by others, with the sense of letting someone down, of not living up to shared standards or internalized ideals. While these emotional responses are related, and often occur in tandem, they are usefully distinguished from one another.^18^ As author-physician Aaron Lazare writes, "guilt usually attaches to a specific instance of wrongdoing towards another …[,] whereas shame appears to be a response to a more general judgment about the self."^19^ When shame and guilt are differentiated in this way, then the common response to guilt is "to make amends," while the common response to shame is "to hide—to avoid contact or to turn away."^20^

While shame and guilt can be conceptually differentiated, they are also experienced differently. Guilt involves a negative feeling that arises upon reflection of a wrongdoing or transgression, and can be discharged or dissolved through apology or reparations; it can be overcome and forgotten. Conversely, shame does not require reflection on one's wrongdoing to be experienced. Instead, shame reactions "can be unexpected, jarring experiences" that occur before any opportunity for thought or reflection,^21^ making it a difficult and even devastating emotion that is not easily discharged or dissipated. Shame can last a lifetime and burns brightest in our memories, ready to resurface, and to be relived, at any moment.^22^ As Ofri notes in her discussion of shame in medical practice: "Shame worms its way into the heart and is remembered like few other things."^23^ A shame event can go to the core of an individual and their identity, making them feel perpetually exposed, inferior, and deficient.^24^ For the psychologist Silvan S. Tomkins, this "inner torment" of shame can make one feel "naked, defeated, alienated, lacking in dignity or worth."^25^ Gershan Kaufman echoes this observation, describing shame as a "wound made from[Other P-330] the inside by an unseen hand" which leads to feeling "fundamentally deficient as individuals, diseased, defective."^26^ To experience shame is "to experience the very essence or heart of the self as wanting. Shame is inevitably alienating, isolating, and deeply disturbing."^27^ Shame, as these descriptions demonstrate, can be powerful and painful and reach to the core of our self and self-conception. When faced with shame, common reactions include "hiding," "escaping," "disappearing from view" and "shrinking into the floor."^28^ Withdrawing and distancing oneself (both physically and psychologically) from a shame event is, then, a common coping strategy.

Despite being differentiated both conceptually and experientially, it must be emphasized that shame and guilt are hard to disentangle; they often become conflated or confused. Or as Lazare simply notes, in his discussion of shame and guilt as fundamental motivations for apologies (especially in medical contexts), "we often experience them simultaneously."^29^ Helen Block Lewis gives more nuance to this phenomenon in her psychoanalytic account: "shame and guilt may function sequentially or as 'defenses' against each other. One may, for example, feel ashamed of some failure in achievement and in the next moment feel guilty for caring about success. Or one may feel guilty for some moral lapse and remain ashamed of 'moral weakness' long after the specific lapse has been forgotten."^30^ In fact, when shame and guilt are both present, Lewis argues, individuals often suppress intense shame (the more difficult experience) and, instead, articulate guilt experiences (which can be dealt with more readily).

## III. Shame and Guilt in Accounts of Medical Error: Danielle Ofri and *State of Wonder*

The complex relationship between shame and guilt plays out explicitly in narrative accounts of medical error in medical confessional and memoir writing—literature that is, in part, focused on recounting and sharing the emotional and personal impact of medical practice and being part of the medical profession.^31^ Ofri writes of her own experience of making a mistake when she was a second-year resident in a busy emergency room. Written almost twenty years after the incident took place, Ofri's account is an honest and powerful portrait of the affective toll of shame and guilt for the "second victim," while also vividly rendering the difficulties of facing up to one's shame. She writes: "I found it extraordinarily difficult to put those words to[Other P-331] paper. … [T]urning out the layers of shame into the bright light, was a palpable agony."^32^

Ofri's patient, a prisoner who had been unable to access insulin, was brought in with DKA—diabetic keto-acidosis. Although her initial management of his case was successful, Ofri then made an error and "proceeded to nearly kill … [the] patient."^33^ Once she realized that the patient was in peril, Ofri called the Senior Medical Resident for help:

"What were you thinking?" the senior resident said, her normally pleasant voice now like a drill sergeant's.

I stood there stone still as my brain cells slowly dissolved into muck.

"What were you thinking?" she repeated, her voice now thundering through the ER, despite the pandemonium swelling around us. Lives were at stake, left and right, and she clearly wasn't going to let me get out of this.

I couldn't even muster a whisper. … I felt a gulf widen around me, as though I'd just lost control of my bladder and was standing in a growing puddle of mortification.^34^

Shame set in as Ofri was questioned repeatedly in front of the intern she had been mentoring: "I could almost feel myself dying away on the spot. In fact, for many minutes, that seemed preferable. … I wanted to evaporate, to disappear, to expire from that horrific moment of shame."^35^ Ofri inflects her experience of shame with the nuance found in the health research literature by explicitly drawing on Lazare's distinction between guilt and shame to frame it:

When I think back to that moment in the ER when the senior resident berated me for my error, it was not guilt but shame that overpowered me. Of course I felt guilty—that was the easy part. I had no trouble berating myself for the error. But it was the shame that was paralyzing. It was the shame of realizing that I wasn't who I thought I was, that I was not who I'd been telling my patient and my intern I was. … It was that up until that moment, I'd thought I was a competent, even excellent, doctor. In one crashing moment of realization, that persona shattered to bits.^36^

This "shattering" experience, Ofri makes clear, had long-term consequences, for her teaching style and for her personal life: "I spent many weeks afterward flagellating my brain for its incompetence, berating[Other P-332] myself for my idiocy."^37^ Many years later, when pregnant, she bumped into the chief resident when they attended the same antenatal clinic. While they chatted easily about non-medical things, Ofri still sensed a residual impact from that event: "I doubted she even remembered. … But for me, the shame of my error and the resultant loss of self-esteem would not release their grip."^38^ Ofri closes her account with an appeal to medical professionals to tend to their "inner landscapes"—their guilt-shame response—to facilitate more open discussion about medical error.

Ofri's essay invites a parallel reading of Marina's experience in *State of Wonder*. Like Ofri's, Marina's shame at her error is compounded, even exceeded, by her concern at failing in the eyes of a respected supervisor and mentor. Marina idealizes Swenson, a formidable, elusive and no-nonsense clinical teacher. A conversation with Anders shortly before his departure for Manaus reveals this ideation not simply as a lingering effect on Marina, many years later, but also as a well-worn character type associated with medical schools:

"What is she like, anyway?" Anders asked her two or three days before he left.

Marina took a moment. She saw her teacher down in the pit of the lecture hall, observed her at a safe and comfortable distance. "She was an old-style medical school professor."

"The stuff of legends? A suicide in every class?"

All she said then was, "Yes."

(30–31)

Her obsessive preoccupation that she has failed to live up to Swenson's ideals and standards is at the nub of her shame: "[she] played this film in her head every hour, waking and sleeping. … She slowed down the tape to a crawl. She looked at every frame separately. …

[S]he was terrified that she was doing something wrong in the eyes of Dr. Swenson."^39^

Critically, however, the usually reciprocal relation described by Ofri, between experiencing shame (with its attendant bodily sensations) and being shamed (as an interpersonal act of coercion), is here absent. This does not mean, however, that the experience of shame is any easier to deal with. As the health research literature demonstrates, supervisors play a critical role in how a medical learner experiences shame, and whether a shame reaction is amplified or mitigated.^40^ Mara Lynne Zabari and Nancy Southern's study of error reporting among obstetric clinicians demonstrates the central role that managers or supervisors[Other P-333] play in producing either a psychologically safe or threatening environment after a medical error.^41^ When a supervisor is approachable, accessible, and willing to share their own experiences of error, errors can be framed as learning opportunities. In contrast, when there is no support after a medical error, experiences of "abandonment" follow.^42^

After the accident Swenson never speaks to Marina about what had transpired: "Marina was a sinking ship and from the safety of dry land Dr. Swenson turned her back and walked away" (70). In Ofri's case, her senior resident "graduated and went off to another job," without any suggestion of a rapproachement or debrief.^43^ Such a lack of support after a medical error makes it more likely that physicians will feel "unworthy and incompetent."^44^ In these cases, individuals feel their environment is too risky to restore their self-image and instead attempt to remove themselves from the situation, either psychologically or physically.^45^

Withdrawal is precisely the response Marina chooses. As a result of the incident, Marina changes her profession; after finishing four years of a five-year program in obstetrics, she switches to clinical pharmacology and enrolls in a three-year PhD program. She feels she has failed as a doctor, so she becomes a good researcher, eventually moving to Minnesota to take a position at Vogel. At the same time, as part of the fallout from the accident, she leaves her husband. Marina puts distance between herself and everyone who knew about her former identity: "The people who did know the details of what had happened … one by one she found a way not to know them anymore. She no longer knew Dr. Swenson."^46^

While Marina articulates the emotional landscape driving these drastic life changes as "a great, lumbering guilt," reading the fallout of this event alongside Ofri's own account helps us see that Marina's feelings of guilt are, indeed, the easy part. It is in fact the shame of not being "who I thought I was" that proves ontologically destabilizing and provokes Marina's complete withdrawal from her former life. In short, guilt is insufficient to explain Marina's decision to leave medicine, change careers, divorce her husband, move across the country, and spend years obsessing over her medical error and Swenson's response.

While shame is never explicitly identified as part of Marina's experience, what is evident in Marina's story is a medical learner struggling to comes to terms with a sentinel emotional event: the deep shame that she feels as a result of medical error that was subsequently handled badly by her immediate supervisor and respected mentor. Following Lewis's psychoanalytic account regarding the covering over[Other P-334] of shame by guilt, it seems clear that Marina's own shame becomes obscured by guilt as a coping mechanism. Marina's (unarticulated) shame comprises three sequential shame triggers reported by medical learners: first, a significant event that caused a patient unnecessary harm; second, the fear of judgment from an esteemed supervisor and mentor; third, callous treatment from a supervisor after the incident.^47^

## IV. What Can *State of Wonder* Help to Understand about Shame in Medical Error?

Although Marina's medical error plays a crucial role in the narrative, it has been largely overlooked in critical responses to the novel. These have tended to focus on its reproduction of the "jungle medicine narrative," wherein pristine tropical forests, populated by wise native people, house rare and potentially life-saving medicinal plants waiting to be "discovered" by Western saviors.^48^ These precious plants are threatened by the twinned forces of immediate extinction as a result of deforestation, and more gradual exploitation by foreign pharmaceutical companies.^49^ It is telling, then, that one of the few reviews to focus on Marina's medical error was written by Ofri in 2011, some two years before she republished her 2010 essay on shame and medical error as a chapter in *What Doctors Feel*.

In Ofri's review she repeatedly, and unfavorably, compares the novel to spy novels ("James Bond-like drama"; "Cold War thriller"), suggesting that "for high drama, pharmaceuticals and their vicissitudes don't quite cut it, even if you stick them in the loneliest outpost of the Amazon."^50^ Ofri frames the comparison to Conrad in similarly deprecating terms. She writes: "Despite carefully crafted prose, none of [Marina's] misadventures have the sizzle of Joseph Conrad's *Heart of Darkness*, which is manifestly beating in the background of Patchett's novel."^51^ Ofri's concern with the novel's dramatic failings is more interesting when aligned with her references to its treatment of medical error, where "as is often the case, the senior physician abandoned the junior physician in the face of medicolegal calamity, and we know who was left to face the flames." According to Ofri, "Even this human element doesn't offer much resonance," since "physicians who have experienced medical error and the devastating, lasting emotional ramifications, will find that this portrayal does not come close to the real McCoy. Adding novelistic drama to a medical error is wholly unnecessary, since real errors tend to be sufficiently dramatic—at least to the involved parties—on their own."^52^[Other P-335]

In a clear spoiler (and with significant understatement), Ofri addresses one of the book's pivotal moments: Swenson's revelation that she has become pregnant at the age of 72, and that she needs Marina to perform an emergency C-section, viscerally revisiting and resolving the scene of the shame that has plagued Marina for years. As Ofri dryly observes, this "is not how most medical errors get worked out."^53^ There is a clear tension in the review between the failure of the novel as a pharmaceutical thriller—its lack of "sizzle"—and its overwriting of medical error with "wholly unnecessary" drama.

Ofri is perhaps right to criticize the novel's unsatisfying resolution to the problem of shame. However, the novel's failure to match up to the spy novels of Ofri's comparison actually serves to illustrate how "the devastating, lasting emotional ramifications" of medical error register across a life course that is not bounded by the discrete spaces generally associated with the medical profession. Ofri's account of her own sentinel emotional event provides a useful frame for reading Marina's. Reading the two together allows us to elaborate on the novel's references to guilt, finessing out of them a more nuanced acknowledgement of shame.

As Marina's story makes clear, the shame arising from a medical error that has not been well managed by immediate supervisors or the institutional environment can inflict long-term damage. This threatens one's "sense of belonging within the profession," as William Bynum and colleagues postulate.^54^ But it can also shape much that lies beyond the profession. *State of Wonder* illustrates the consequences of shame through a lush narrative that spans personal, social, institutional, and political registers. In Ofri's writing, we find a rich and nuanced autobiographical account of medical shame, but also one that is neatly confined to the professional setting, as though medical shame only has consequences within the walls of the hospital. The consequences of Ofri's shame are articulated in precisely this way: "Lord knows I will never take DKA for granted. If so much as a single cc of an insulin drip is running into the veins of one of my patients, I am hovering like a hawk, compulsively checking and rechecking every minutia of lab data. … To this day, when I teach my students about DKA, I emphasize that clinical point with the vehemence of Moses on Sinai: 'Thou shalt not turn off the insulin drip until long-acting insulin hath been administered.'"^55^ Ofri does reflect on the multifaceted consequences of shame arising from medical error in the areas of medical protocol and pedagogy, as well as how such consequences figure in some research within academic medicine. Yet her own personal shame experience lacks[Other P-336] these broader dimensions. What remains underemphasized in Ofri's personal account of her shame are not only social, institutional, and political registers, but the human outside of the white coat. Linda Gask identifies this as the point of medical memoir writing: "the continued struggle to manage the impact of the profession of medicine on one's own humanity."^56^ *State of Wonder* makes connections that Ofri's essay gestures towards, without fully articulating.

To reread Ofri's essay in light of the novel's focus on extended fertility invites interpretations of situational irony: Ofri's subsequent encounter with her chief resident occurred when they were both "hulkingly pregnant."^57^ She describes the encounter as "ironic, even prophetic," because it brings them together as expectant mothers rather than doctors, albeit in a healthcare setting (the antenatal clinic), but she does not explain why this might be either ironic or prophetic.^58^ The implication is that the encounter, though pleasant, was not reparative; the shame that Ofri experiences is not alleviated by the chief resident, who cannot acknowledge what she does not remember. In contrast, towards the end of *State of Wonder*, Marina does receive the absolution of her revered teacher, Swenson. Not only does Swenson remember the accident, she offers the reassurance Marina had been missing all those years ago—the support of a supervisor-mentor that could have mitigated Marina's shame. Swenson reassures Marina that she merely made a mistake as a doctor (a fact deserving of a guilt response), rather than Marina's own conclusion, that she was mistaken to want to be a doctor (an idea arising from her shame response). Swenson states the matter plainly: "You made a very common mistake that night at the General. You rushed. Nothing more than that. Had it not been the eye you would have forgotten all about it in a week. … In retrospect the real loss was your quitting the program" (356). Swenson's casual revelation is so unexpected that it causes Marina to sit down in surprise: "and there it went, the burden of her lifetime, taken" (356).

This implausible resolution, which Ofri treats so scathingly in her review of the novel, takes place when Marina wavers over performing the C-section on Swenson, who has impregnated herself at age 72 to serve as the first human trial of the fertility drug that she has been developing for Vogel. Relieving Marina of her "burden" is not simply a post-hoc exoneration, but a visceral reenactment of the conditions under which the original shaming happened. Ofri's negative response to this fictional turn of events is, of course, aesthetically justified: the resolution is too swift, too easy. But it also reflects, perhaps,[Other P-337] Ofri's frustration at seeing a moment of great personal pain blithely resolved in an encounter that so closely paralleled her own. After all, Ofri describes the "drawing forth of those emotions" as "exhausting": "there was no magical resolution."^59^ Read alongside Ofri's memoir, the resolution in *State of Wonder* becomes too neat, eliding the inevitable emotional complexity that follows sentinel emotional events.

Ofri's encounter with her chief resident was ironic or prophetic not merely because they were not performing their roles as doctors; when they met, they were preparing for their roles as mothers. In this regard, *State of Wonder* does address a concern latent, if unexamined, in Ofri's essay: the way that shame about medical error seeps into other, apparently unrelated, areas of a person's life. Marina reconfigures her life around the impact of her sentinel event, which is linked to a set of voided conversations with her own mother:

Marina didn't tell her what it was she was actually breaking up with. She didn't tell her that the life she had ruined was not her own nor [her ex-husband] Josh Su's but someone else's, someone she didn't even know. She did not tell her mother about the accident, nor about the Spanish Inquisition that had followed. She did not tell her about the switch to pharmacology and then she mentioned it so casually that it seemed like the most natural thing in the world. She did not tell her mother about Dr. Swenson.

(61–62)

This repetition of statements in the apophatic form, "did not tell," develops what we might call a series of pregnant silences around the sentinel emotional event, and also through its ripple effects on Marina's marriage, career change, and relationship with her mother. The novel's resolution is not just the lifting of this burden because Anders has been found alive and Marina may or may not be pregnant with his child. Patchett exploits the same trope that makes Ofri's encounter so "prophetic": the association of pregnancy with new life and new possibility.

## V. Writing Shame and the Shame of Writing

In *State of Wonder*, we contend, shame is both a subterranean force driving the protagonist and a core feature of the novel's structure. So why does Marina render her affective fallout not as "shame" but as "a great lumbering guilt"? Following Lewis, this conspicuous[Other P-338] absence points to the tendency for shame to be absorbed by guilt, or other responses, in our lived experience. But it also raises the issue of "writing shame": the dual difficulty of acknowledging shame and subsequently accounting for it through writing.

Shame is characterized by concealment and secrecy. As noted above, it is so painful and threatening that it is often bypassed or repressed for other experiences.^60^ For instance, the relational psychotherapist Patricia DeYoung discusses how shame often "disappears."^61^ "Instead of feeling the emotional intensity of shame itself," she writes, "a client may turn either to obsessive self-hatred or obsessive thoughts about what went wrong in interactions between self and others."^62^ As Sandra Lee Bartky notes in her phenomenological discussion of shame in women's experience, some individuals may never identify their experience as containing shame and instead simply experience personal inadequacy and low self-regard.^63^ DeYoung's analysis of her clients' experiences concurs: she argues that some of her clients who suffer from chronic shame do not even know that they are experiencing it (and related strategies to circumvent the threat of shame) with debilitating frequency.

On a subjective level, shame is such a difficult emotion to confront and acknowledge because revealing that one is experiencing shame is *itself* shameful.^64^ As a result, shame can provoke a spiral or "loop,"^65^ where shame incites more shame (about the shame). Shame, as such, is an iterated emotion; its occurrence leads to an intensification or multiplication of itself.^66^This "second-order" shame results from the anxiety it prompts.^67^ As Elspeth Probyn simply notes in her essay "Writing Shame": "shame is a painful thing to write about."^68^

Regarding her own incident, Ofri recalls that it took her nearly two decades to write about what happened, and even then found it an "agony" to describe. While there was "no magical resolution," Ofri notes, the writing "gave me the chance to scrabble about in the mud with the emotions and arrive at a stalemate of sorts."^69^ What Ofri describes is not simply a referential examination of shame in her writing. It includes a reflexive shame brought about by the writing process itself.^70^

Ofri's reflection on the writing process might well explain why she chooses the unusual adjective "sizzle" to describe Conrad's style in *Heart of Darkness*. Since Conrad's style can hardly be said to sizzle, we might think of Ofri's sizzle as a reference to something visceral: the evocation of an embodied confusion that covers over a burning[Other P-339] shame. In Conrad's case, the layered narration may serve as a means to shield the author from the shame of the colonial project, in particular his implication within the multiple shameful injustices recounted by Marlow. Edward Said, writing of *Heart of Darkness*, noted that we might read the obscurity in Conrad's writing style as just such an avoidance tactic: his "obscurity … is a function of secret shame. Paradoxically, however, the secret is all too easily prone to the wrong kind of exposure, which Conrad's notoriously circumspect methods of narrative attempt to forestall. The reflective narrator is always a narrator preventing the wrong sort of interpretation."^71^ While recognizing the importance of Said's insight, Timothy Bewes suggests it is better inverted: "Conrad's shame is not a 'secret' underlying the obscurity, but the opposite: a function of manifestation. Conrad's shame is not secret but overt; he is a writer for whom writing cannot *not* be shameful."^72^ Obscurity does not cover over the shame of writing; rather, we might read obscurity as its manifestation, its evidence. In this regard, we might find that the clarity of Ofri's writing presents a refreshing interruption of shame's obscurity, or shame's tendency toward concealment and secrecy. Ofri writes through the pain of revealing her shame, and lays it out in plain view.

In contrast, *State of Wonder* fastidiously conceals Marina's shame. While the novel's clear presentation of Marina's inner torment demonstrates all the structural elements of shame (psychological and physical withdrawal, experience of personal inadequacy, concealment, intense self-consciousness, remorse, obsessive memories, "the burden of her lifetime"), it remains buried in the narrative, surfacing only inferentially with reference to a "lumbering guilt." *State of Wonder*'s untroubled prose, so different from Conrad's obscurity, does not expose shame; rather, we might interpret the prose's lack of trouble as the ultimate "defensive script" to bypass the threat of shame, and, at the same time, the threat of writing about it.^73^ In this way, the novel precisely enacts one of shame's core phenomenological features: it is not experienced as present, and instead experienced as a "present absence."^74^ It may indeed be the novel's failure to engage fully with Marina's shame, *as shame*, that so provoked Ofri. By adding "novelistic drama" to the medical error, the novel leaves out its most dramatic element: the anxiety about shame that constricts the writer's pen, that circumscribes its product.[Other P-340]

## VI. Conclusion

Whereas Ofri engages explicitly with shame, *State of Wonder* does not. The novel's structure suggests, however, that this occlusion is a semantic matter: shame is encoded within the narrative, even if it dare not speak its name. The elaboration begs the question, then, why read the novel for an account of shame and medical error, rather than Ofri's essay "Ashamed to Admit It," or her subsequent nonfiction work, *What Doctors Feel*? Given the sophistication of Ofri's argument, her writing serves as a more useful tool to break apart fused accounts of guilt-shame in medical error. Moreover, implicit in Ofri's agonistic relation to the novel is the suggestion that only writing that fully reprises shame's contortions, whether through its descriptions (Ofri) or its form (Conrad), can usefully or accurately depict it. This fails to appreciate *State of Wonder*'s true contribution, as an examination of shame's more lasting consequences over a life course. *State of Wonder* illustrates the complex and long-term consequences of experiencing shame as a sentinel emotional event: how these events can shape medical professionals over the course of their professional lives, and beyond. In this respect, Patchett's refusal, or failure, to engage more directly with shame at the scene of writing enables her to engage more fully with what shame does. Ultimately, attending to the novel's failure, what Ofri identifies as a trite solution to Marina's chronic anxiety threatens to obscure from interpretation its achievement, a rare consideration of how medical professionals deal with sentinel events as a regular feature of their lives.

## NOTES

1
Wu, "Medical Errors."

2 Fletcher, *Myths of the Underworld in Contemporary Culture*.

3 Bynum et al., "Sentinel Emotional Events," 90.

4 Ofri, *When We Do Harm*, 3.

5 Robertson and Long, "Medicine's Shame Problem," 331. See also Peters and King, "Perfectionism in Doctors."

6 Bynum and Goodie, "Shame, Guilt and the Medical Learner," 1046.

7 Ofri, *What Doctors Feel*, 129.

8 Aubin and King, "Healthcare Environment," 31.

9 While this seems self-evident, it was only with the publication of the Institute of Medicine report *To Err Is Human* in 1999 that there was widespread acknowledgment of the consequences of medical error for both patients and practitioners, along with explicit reporting of the high incidence and substantial financial cost of medical mistakes globally. See also Nydoo et al., "Second Victim Phenomenon," 3.

10 Ann Patchett, *State of Wonder*, 69. Further references will be cited parenthetically in the text.

11
Wu, "Medical Errors."

12 Nydoo et al., "Second Victim Phenomenon," 2.

13
Zabari and Southern, "Effects of Shame and Guilt."

14 Aaron Lazare, in his seminal 1987 article "Shame and Humiliation in the Medical Encounter," makes the point that clinicians can experience shame and humiliation in relation to not performing a therapeutic procedure according to one's own or one's colleagues' standards (Lazare, "Shame and Humiliation," 1656). Interestingly, Lazare's formulation of shameful events for clinicians, coming over a decade before the release of the report *To Err Is Human*, does not acknowledge the idea that clinicians can make mistakes, nor that they can also be "victims" of a medical error.

15 Bynum and Goodie, "Shame, Guilt and the Medical Learner," 1047.

16 Aubin and King, "Healthcare Environment," 31.

17 While there is limited empirical evidence on experiences of shame and guilt in medical error, there are some preliminary studies which support the anecdotal evidence. See, for example, Bynum and Goodie, "Shame, Guilt and the Medical Learner."

18 There are many competing accounts about how to distinguish shame and guilt within philosophy, psychology, and emotion research. These debates are beyond the concerns of this work, but if they are of interest, see for instance Teroni and Deonna, "Differentiating Shame from Guilt."

19 Lazare, *On Apology*, 136. See also Karlsson and Sjoberg, "Experiences of Guilt and Shame."

20 Lazare, *On Apology*, 136.

21 Bynum et al., "Sentinel Emotional Events," 90.

22 See Dolezal, *The Body and Shame*.

23 Ofri, *What Doctors Feel*, 130.

24 See Dolezal, "Phenomenology of Shame."

25 Tomkins, *Affect, Imagery, Consciousness*, 118.

26 Kaufman, *Psychology of Shame*, 5, 18.

27 Kaufman, *Psychology of Shame*, 18.

28
Dickerson, Gruenewald, and Kemeny, "When the Social Self."

29 Lazare, *On Apology*, 136.

30 Lewis, *Shame and Guilt in Neurosis*, 27.

31
Lyons, Gibson, and Dolezal, "Stories of Shame."

32 Ofri, *What Doctors Feel*, 138.

33 Ofri, *What Doctors Feel*, 124.

34 Ofri, *What Doctors Feel*, 126.

35 Ofri, *What Doctors Feel*, 130.

36 Ofri, *What Doctors Feel*, 129.

37 Ofri, *What Doctors Feel*, 128.

38 Ofri, *What Doctors Feel*, 138.

39 Patchett, *State of Wonder*, 68.

40 Bynum et al., "Sentinel Emotional Events," 89.

41
Zabari and Southern, "Effects of Shame and Guilt."

42 Robertson and Long, "Medicine's Shame Problem," 332.

43 Ofri, "Ashamed to Admit It," 1550.

44 Robertson and Long, "Medicine's Shame Problem," 332.

45 See de Hooge, Zeelenberg, and Breugelmans, "Functionalist Account."

46 Patchett, *State of Wonder*, 64–65.

47
Bynum et al., "Sentinel Emotional Events."

48 Voeks, *Ethnobotany of Eden*, 4.

49 Voeks, *Ethnobotany of Eden*, 4.

50 Ofri, "Pharma in the Jungle," 1289.

51 Ofri, "Pharma in the Jungle," 1289.

52 Ofri, "Pharma in the Jungle," 1289.

53 Ofri, "Pharma in the Jungle," 1289.

54 Bynum et al., "Sentinel Emotional Events," 90.

55 Ofri, *What Doctors Feel*, 139, 30.

56 Gask, "I Remember, I Remember," 472.

57 Ofri, *What Doctors Feel*, 138.

58 Ofri, *What Doctors Feel*, 138.

59 Ofri, *What Doctors Feel*, 138.

60 See, for example, Stephen Pattison, *Shame*; Donald Nathanson, *Shame and Pride*; Helen B. Lewis, *Role of Shame in Symptom Formation*.

61 DeYoung, *Understanding and Treating Chronic Shame*, 24.

62 DeYoung, *Understanding and Treating Chronic Shame*, 24, 4.

63 Bartky, *Femininity and Domination*, 85.

64 The shamefulness of shame can vary for certain groups. For example, it is suggested by Aneta Stepien that shame is particularly shameful for men. As a result they are much more likely to repress, hide, or deny shame, perhaps bypassing it for other emotions or experiences such as depression or anger. See Stepian, "Understanding Male Shame."

65 Scheff, "Shame and the Social Bond," 90.

66 Lewis, *Shame and Guilt in Neurosis*, 202. See also Kaufman, *Psychology of Shame*, 4, 20.

67 Lee and Wheeler, *Voice of Shame*, vii.

68 Probyn, "Writing Shame," 130.

69 Ofri, *What Doctors Feel*, 139.

70 For an account of the reflexive potential in writing shame see Shiels and Walsh, "Introduction: Shame and Modern Writing."

71 Said, quoted in Bewes, *Event of Postcolonial Shame*, 95.

72 Bewes, *Event of Postcolonial Shame*, 93.

73 For more on "defensive scripts," see Nathanson, *Shame and Pride*.

74 See Dolezal, "Horizons of Chronic Shame."

## References

[b1] Aubin Diane, King Sharla (2018). The Healthcare Environment: The Perfect Ecosystem for Growing Shame. Organizational Culture.

[b2] Bartky Sandra Lee (1990). Femininity and Domination: Studies in the Phenomenology of Oppression.

[b3] Bewes Timothy (2011). The Event of Postcolonial Shame.

[b4] Bynum William E., Artino Anthony R., Uijtdehaage Sebastian, Webb Allison M. B., Varpio Lara (2019). Sentinel Emotional Events: The Nature, Triggers and Effects of Shame Experiences in Medical Residents. Academic Medicine.

[b5] Bynum William E., Goodie Jeffrey L. (2014). Shame, Guilt, and the Medical Learner: Ignored Connections and Why We Should Care. Medical Education.

[b6] DeYoung Patricia A. (2015). Understanding and Treating Chronic Shame: A Relational/Neurobiological Approach.

[b7] Dickerson Sally S., Gruenewald Tara L., Kemeny Margaret E. (2004). When the Social Self Is Threatened: Shame, Physiology, and Health. Journal of Personality.

[b8] Dolezal Luna (2015). The Body and Shame: Phenomenology, Feminism and the Socially Shaped Body.

[b9] Dolezal Luna (2002). The Horizons of Chronic Shame. Human Studies.

[b10] Dolezal Luna (2015). The Phenomenology of Shame in the Clinical Encounter. Medicine, Healthcare, and Philosophy.

[b11] Fletcher Judith (2019). Myths of the Underworld in Contemporary Culture: The Backward Gaze.

[b12] Gask Linda (2019). I Remember I Remember: The Therapeutic Power of the Medical Memoir. Lancet Psychiatry.

[b13] Hooge Illona E. de, Zeelenberg Marcel, Breugelmans Seger M. (2011). A Functionalist Account of Shame-Induced Behaviour. Cognition and Emotion.

[b14] Institute of Medicine (1999). To Err Is Human: Building a Safer Healthcare System.

[b15] Karlsson Gunnar, Sjoberg Lennart Gustav (2009). The Experiences of Guilt and Shame: A Phenomenological-Psychological Study. Human Studies.

[b16] Kaufman Gershen (1993). The Psychology of Shame: Theory and Treatment of Shame Based Syndromes.

[b17] Lazare Aaron (2004). On Apology.

[b18] Lazare Aaron (1987). Shame and Humiliation in the Medical Encounter. Archives of Internal Medicine.

[b19] Lee Robert G., Wheeler Gordon (1996). The Voice of Shame: Silence and Connection in Psychotherapy.

[b20] Lewis Helen B. (1987). The Role of Shame in Symptom Formation.

[b21] Lewis Helen Block (1971). Shame and Guilt in Neurosis.

[b22] Lyons Barry, Gibson Matthew, Dolezal Luna (2018). Stories of Shame. Lancet.

[b23] Nathanson Donald (1992). Shame and Pride: Affect, Sex and the Birth of the Self.

[b24] Nydoo Puvashnee, Pillay Basil J., Naicker Thajasvarie, Moodley Jagidesa (2019). The Second Victim Phenomenon in Health Care: A Literature Review. Scandinavian Journal of Public Health.

[b25] Ofri Danielle (2010). Ashamed to Admit It: Owning up to Medical Error. Health Affairs.

[b26] Ofri Danielle (2011). Pharma in the Jungle. Lancet.

[b27] Ofri Danielle (2013). What Doctors Feel: How Emotions Affect the Practice of Medicine.

[b28] Ofri Danielle (2020). When We Do Harm: A Doctor Confronts Medical Error.

[b29] Patchett Ann (2012). State of Wonder.

[b30] Pattison Stephen (2000). Shame: Theory, Therapy, Theology.

[b31] Peters Mike, King Jenny (2012). Perfectionism in Doctors. British Medical Journal.

[b32] Probyn Elspeth (2005). Writing Shame. Blush: The Faces of Shame.

[b33] Robertson Jennifer J., Long Brit (2019). Medicine's Shame Problem. Journal of Emergency Medicine.

[b34] Scheff Thomas J. (2000). Shame and the Social Bond: A Sociological Theory. Sociological Theory.

[b35] Shiels Barry, Walsh Julie, Shiels Barry, Walsh Julie (2018). Introduction: Shame and Modern Writing.

[b36] Stepian Aneta (2014). Understanding Male Shame. Masculinities: A Journal of Identity and Culture.

[b37] Teroni Fabrice, Deonna Julien (2008). Differentiating Shame from Guilt. Consciousness and Cognition.

[b38] Tomkins Silvan S. (1963). Affect, Imagery, Consciousness: The Negative Affects.

[b39] Voeks Robert A. (2018). The Ethnobotany of Eden: Rethinking the Jungle Medicine Narrative.

[b40] Wu A. W. (2000). Medical Errors: The Second Victim. The Doctor Who Makes the Mistake Needs Help Too. British Medical Journal.

[b41] Zabari Mara Lynne, Southern Nancy L. (2018). Effects of Shame and Guilt on Error Reporting among Obstetric Clinicians. Journal of Obstetric, Gynecologia and Neonatal Nursing.

